# Support vector machine model for diagnosis of lymph node metastasis in gastric cancer with multidetector computed tomography: a preliminary study

**DOI:** 10.1186/1471-2407-11-10

**Published:** 2011-01-11

**Authors:** Xiao-Peng Zhang, Zhi-Long Wang, Lei Tang, Ying-Shi Sun, Kun Cao, Yun Gao

**Affiliations:** 1 Department of Radiology, Key laboratory of Carcinogenesis and Translational Research (Ministry of Education), Peking University Cancer Hospital & Institute, (No.52, Fucheng Road, Haidian District), Beijing, (100142), China

## Abstract

**Background:**

Lymph node metastasis (LNM) of gastric cancer is an important prognostic factor regarding long-term survival. But several imaging techniques which are commonly used in stomach cannot satisfactorily assess the gastric cancer lymph node status. They can not achieve both high sensitivity and specificity. As a kind of machine-learning methods, Support Vector Machine has the potential to solve this complex issue.

**Methods:**

The institutional review board approved this retrospective study. 175 consecutive patients with gastric cancer who underwent MDCT before surgery were included. We evaluated the tumor and lymph node indicators on CT images including serosal invasion, tumor classification, tumor maximum diameter, number of lymph nodes, maximum lymph node size and lymph nodes station, which reflected the biological behavior of gastric cancer. Univariate analysis was used to analyze the relationship between the six image indicators with LNM. A SVM model was built with these indicators above as input index. The output index was that lymph node metastasis of the patient was positive or negative. It was confirmed by the surgery and histopathology. A standard machine-learning technique called k-fold cross-validation (5-fold in our study) was used to train and test SVM models. We evaluated the diagnostic capability of the SVM models in lymph node metastasis with the receiver operating characteristic (ROC) curves. And the radiologist classified the lymph node metastasis of patients by using maximum lymph node size on CT images as criterion. We compared the areas under ROC curves (AUC) of the radiologist and SVM models.

**Results:**

In 175 cases, the cases of lymph node metastasis were 134 and 41 cases were not. The six image indicators all had statistically significant differences between the LNM negative and positive groups. The means of the sensitivity, specificity and AUC of SVM models with 5-fold cross-validation were 88.5%, 78.5% and 0.876, respectively. While the diagnostic power of the radiologist classifying lymph node metastasis by maximum lymph node size were only 63.4%, 75.6% and 0.757. Each SVM model of the 5-fold cross-validation performed significantly better than the radiologist.

**Conclusions:**

Based on biological behavior information of gastric cancer on MDCT images, SVM model can help diagnose the lymph node metastasis preoperatively.

## Background

Gastric cancer is one of the leading causes of cancer-related deaths worldwide [1]. Lymph node status is an important prognostic factor regarding long-term survival [2]. The TNM staging system based on American Joint Committee on Cancer (AJCC) is accepted widely now [3]. The 5-year survival rate of patients in the N0 stage after surgery was 86.1%, while the N1, N2, and N3 stage patients dropped to 58.1%, 23.3% and 5.9%, respectively [4].

At present, many imaging techniques have been used to assess gastric cancer, including abdominal ultrasound, endoscopic ultrasound (EUS), multi-slice spiral CT, conventional MRI, and FDG-PET. However, these imaging methods cannot reliably confirm or exclude the presence of lymph node metastasis [1]. A meta-analysis showed that the average sensitivity and specificity in determining LN metastasis were as follows: 39.9% and 81.8% for abdominal ultrasound, 70.8% and 84.6% for endoscopic ultrasonography, 80.0% and 77.8% for MDCT, 68.8% and 75.0% for conventional MRI, 34.3% and 93.2% for FDG-PET, and 54.7% and 92.2% for FDG-PET/CT [2]. Any single application of these imaging tools cannot satisfactorily assess the gastric cancer lymph node status. The reason is that we mainly diagnose LNM by the size of lymph nodes. The diagnostic criteria range from 5 mm to 10 mm [2]. But the large lymph nodes may be caused by inflammation and the small lymph nodes may be metastatic. Many studies have shown that gastric cancer LN metastasis was associated with tumor size, depth of invasion, histological type and pathological lymphatic involvement [5-8]. There is no suitable method to combine lymph node size with the multiple factors described above to make a comprehensive analysis. How to integrate the complex factors affecting lymph nodes and improve the accuracy of diagnosing LNM is the topic of our study.

In the past decade, machine-learning methods, complementary to traditional statistical methods, have been used to predict complex biological phenomena. Support Vector Machine is a new generation of learning algorithms developed on the basis of statistical theory. The SVM algorithm has a strong theoretical foundation, based on the ideas of VC (Vapnik Chervonenkis) dimension and structural risk minimization. It has satisfied accuracy [9]. SVM has been used in some medical applications, mainly in molecular biology and neuroimaging [10-12]. It can be used for classification and regression. Given a set of training examples, each marked as belonging to one of two categories, a SVM training algorithm builds a model that predicts whether a new example falls into one category or the other.

The purpose of this study is to use SVM method to analyze the MDCT imaging information related to the biological behavior of gastric cancer and establish the mathematical models to assess lymph node metastasis preoperatively.

## Methods

### Patients

This retrospective study was approved by our institutional review board. Between April 2006 and September 2008, 368 consecutive patients with newly diagnosed gastric cancer were administered preoperative contrast enhancement abdominal CT examinations and then received the gastrectomy at our hospital. The patients corresponded to the inclusion and exclusion criteria below were included in this study.

#### Inclusion criteria

The patients received radical gastrectomy and D2 lymph nodes dissection. They were preoperatively examined with multi- detector row CT. All patients were confirmed as gastric cancer by postoperative histopathology.

#### Exclusion criteria

The patients received preoperative neoadjuvant therapy. Distant metastasis was found in the preoperative examination or in the operation.

Finally, 175 patients (125 males, 50 females, mean age, 59.8 years; range, 30-85 years) comprised our study population. We obtained informed consent from all selected patients prior to the routine clinical course of CT examinations.

### CT Protocol

MDCT was performed using a 64-detector row CT scanner (LightSpeed 64; GE Healthcare, Milwaukee, Wis). Each patient fasted for more than 8 hours before the CT examination. To enable gastric distention and reduce gastric motility, the patients received 8 g gas-producing crystals orally and an intramuscular injection of 10 mg anisodamine 10-15 minute before the examination. Upper abdominal unenhanced CT scans from the diaphragmatic domes to 2 cm below the lower margin of the air-distended gastric body were acquired with a collimation of 0.625 mm, 120-140 kVp, and 300-350 mAs. Subsequently, a total of 100 ml of iopromide (Ultravist; Schering, Berlin, Germany) was administered intravenously through an 18-gauge angiographic catheter inserted into an antecubital vein at 3 mL/sec by using an automatic injector. Contrast-enhanced CT scans were performed in the arterial phase (30 seconds) and in the portal venous phase (70 seconds). We made the multi-planar reconstruction with the portal venous phase image.

### Image Analysis

Two radiologists, one with 3 yrs and the other with 8 yrs experience in abdominal CT performed image analyses jointly to agreement. If there was disagreement, they consulted with another radiologist who had 20 yrs experience in abdominal CT until agreement was achieved. We measured and counted the six indicators on MDCT images by hands as follows:

#### Tumor maximum diameter

Measure the diameter of gastric cancer in the axial, coronal, and sagittal images based the MPR images. And decide the tumor maximum diameter.

#### Tumor classification

Early gastric cancer or Borrmann classification of advanced cancer in the MPR images was determined.

#### Serosal invasion

Axial and MPR images were simultaneously evaluated to determine the serosal invasion. The entire thickening stomach wall abnormally enhanced and linear or reticular structures in the fatty layer surrounding the stomach indicated serosal invasion [13].

#### Number of lymph nodes

The number of all the visible gastric regional lymph nodes in the MDCT images by groups was counted [14].

#### Maximum lymph node size

The short axis of the largest lymph node detected in CT images was measured.

#### Lymph nodes station

The lymph nodes station with MDCT images based on the Japanese classification of gastric carcinoma was determined [14].

### Support vector machine

Support Vector Machine is a supervised machine learning technique that is widely used in pattern recognition and classification problems. SVM algorithm performs a classification by constructing a multidimensional hyperplane that optimally discriminates between two classes by maximizing the margin between two data clusters. This algorithm achieves high discriminative power by using special nonlinear functions called kernels to transform the input space into a multidimensional space [15]. In this study, a free available SVM software called LibSVM 2.89 was used to generate the SVM model [16]. The input indexes were the six indicators collected on MDCT images above. For these indicators, the measurement data could be entered to SVM model directly. While the count data should be defined as some numbers. For example, positive serosal invasion was defined as 1 and negative was -1. The output index was the lymph node metastasis of the patient. It was confirmed by the surgery and histopathology. If the patient had one or more lymph nodes metastasis, it was considered as positive LNM. We defined the positive LNM as 1 while the negative was -1. We selected the RBF Kernel to build the model. To train and test our SVM model, we used a standard machine-learning technique called k-fold cross-validation. Because the whole sample size of our study was not very large, we used 5-fold cross-validation. The whole data were divided into 5 equal and distinct subsets. Four of these subsets are combined and used for training, and the remaining one set is used for testing. This cross-validation process was repeated 5 times, allowing each subset to serve once as the test data set.

### Statistical Analysis

A univariate statistical analysis using the SPSS/PC+ statistical software package version 11.5 (SPSS Inc, IL, Chicago, USA) was performed to evaluate the differences of six imaging indicators between the patients who had LNM or not. The statistical analysis methods were the Independent-samples T test and Mann-Whitney U test. P < 0.05 was considered significant difference. Receiver operating characteristic (ROC) curve was used to evaluate the diagnostic performance of the SVM model. The Medcalc software version 11.2 (Medcalc, Medcalc Software, Ghent, Belgium) was used to make the ROC curves and compare them. In summary, we averaged the area under the curve (AUC) of the ROC curves of the 5-fold cross-validation. We also counted the means of sensitivity and specificity. To compare with the SVM model, we constructed the ROC curve for radiologist assessment by using maximum lymph node size as criteria to classify the LNM. The sensitivity and specificity of the best cut-off point were counted.

## Results

In these 175 cases, there were 134 cases which had lymph node metastasis and 41 cases had not. Patients' clinicopathological features were detailed at the Table 1. We collected the six indicators on MDCT images. The results of the univariate statistical analysis indicated that the all six indicators including serosal invasion, tumor classification, tumor maximum diameter, number of lymph nodes, maximum lymph node size and lymph nodes station were significant different between the LNM positive and negative group (P < 0.001). The means of tumor maximum diameter, number of lymph nodes, and maximum lymph node size in LNM positive group were 56.6 ± 19.5 mm, 10.0 ± 5.5 mm, and 12 ± 8, respectively. They were all higher than those of LNM negative group (Table 2).

**Table 1:** Patient Characteristics

Clinicopathological features	Value
No. of patients	175
Mean age (y)	59.8(30-85)
Ratio of women to men	50:125
Histopathology	
Adenocarcinoma	173(98.9%)
Well differentiated	6(3.4%)
Moderately differentiated	91(52%)
Poorly differentiated	76(43.5%)
Small cell carcinoma	2(1.1%)
lymph node metastasis	
Positive	134(76.6%)
Negative	41(23.4%)

Note.--Numbers in parentheses are the ranges.

**Table 2:** Patient data: The 6 indicators' data of the MDCT images and the results of univariate statistical analysis.

Patient data	LNM(-)	LNM(+)	P value
Patient number	41/175(23.4%)	134/175(76.6%)	
Measurement data*			
Tumor maximum diameter (mm)	39.0 ± 17.0	56.6 ± 19.5	<0.001
Maximum lymph node size (mm)	6.5 ± 2.8	10.0 ± 5.5	<0.001
Number of lymph nodes	7 ± 4	12 ± 8	<0.001
Count data#			
Serosal invasion			<0.001
Yes	15/175(8.6%)	120/175(68.6%)	
No	26/175(14.8%)	14/175(8%)	
Tumor classification			<0.001
Early gastric cancer	9/175(5.1%)	1/175(0.6%)	
BorrmannI	2/175(1.1%)	0/175	
BorrmannII	3/175(1.7%)	9/175(5.1%)	
Borrmann III	27/175(15.4%)	121/175(69.1%)	
Borrmann IV	0/175	3/175(1.7%)	
Lymph nodes station			<0.001
Station1	29/175(16.6%)	44/175(25.1%)	
Station2	12/175(6.9%)	54/175(30.9%)	
Station3	0/175	36/175(20.5)	

* The value of the measurement data was means ± standard deviation. The p value was from Independent-samples T test.

# The value of the count data was the number of data. The p value was from Mann-Whitney U test.

**Figure 1:**
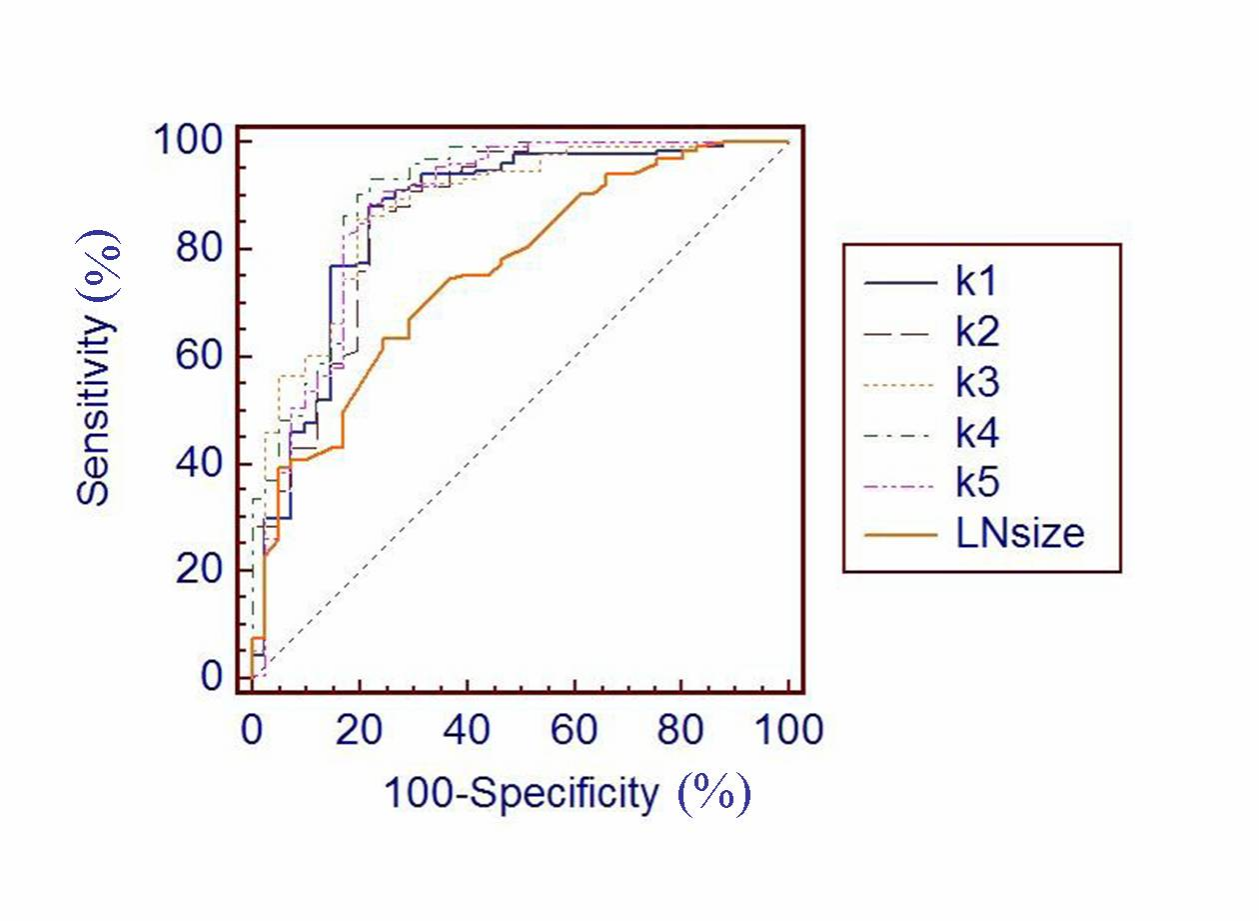
**ROC curve for LNM**. Receiver operating characteristic (ROC) curve for lymph node metastasis with 5-fold cross-validation SVM models and radiologist. The AUC of k1 to k5 SVM models were 0.862, 0866, 0.878, 0.900 and 0876, respectively. Compared with the radiologist, the P values were all less than 0.05 (Table 3). For the five SVM models, the mean of AUCs was 0.876. And the AUC of radiologist based LN size was 0.757.

The radiologist achieved an AUC of 0.757 as classifying lymph node metastasis of the patient by maximum lymph node size. The best cut-off point of maximum lymph node size was 7.7 mm. The sensitivity and specificity were only 63.4% and 75.6%. The SVM's means of the sensitivity, specificity and AUC with 5-fold cross-validation were 88.5%, 78.5% and 0.876, respectively (Table 3). Compared to the radiologist, each AUC of the 5-fold cross-validation SVM models performed significantly better (P < 0.05) than the radiologist (Figure 1,Table 3).

**Table 3:** AUC of SVM model and radiologist

Model	K-fold	Sensitivity	Specificity	AUC*	P value (AUC compared with Radiologist)
SVM	K1	0.881	0.780	0.862 ± 0.038	0.002
	K2	0.866	0.780	0.866 ± 0.037	<0.001
	K3	0.858	0.805	0.878 ± 0.033	<0.001
	K4	0.933	0.780	0.900 ± 0.031	<0.001
	K5	0.888	0.780	0.876 ± 0.038	<0.001
	mean	0.885	0.785	0.876	
Radiologist		0.634	0.756	0.757 ± 0.042	

The sensitivity, specificity and AUC of 5-fold cross-validation SVM models and radiologist for diagnosing lymph node metastasis of patient.

* The value of the data was AUC ± standard deviation.

## Discussion

Lymph node metastasis affects the surgical treatment of patients with gastric cancer and is also an important factor in prognosis. At present, preoperative diagnosis mainly depends on various imaging methods. The standard for judging lymph node metastasis relies on morphological indicators. Lymph node size is the dominant indicator. However, Dorfman RE et al reported that upper limits of normal for lymph node size at abdominal computed tomography varied from 6 to 11 mm [17]. They partly overlapped with the malignant lymphadenopathy. Fukuya T et al showed that CT attenuation and lymph-node configuration could aid in diagnosis of malignant adenopathy [18]. On the contrary, Deutch SJ et al expressed that size, location, contour, density were not helpful in distinguishing benign from malignant lymphadenopathy [19]. Lack of criteria for judging is the main constraint for the prediction of lymph node metastasis preoperatively.

The biological behavior of gastric cancer reflects the histopathological performance of the tumor's malignance and invasion. It affects lymph node metastasis directly or indirectly. The concrete manifestation of the biological behavior includes, for example, tumor size, depth of invasion, tumor invasion of other organs, lymph node metastasis and distant metastasis. MDCT can clearly display these pathological occurrences. Some studies have reported that the accuracy of gastric cancer T staging with MDCT combined with 3D reconstruction was 84-89% [20,21]. Zhang XP et al reported that the number of lymph nodes detected by MDCT showed a significant difference between the lymph node metastasis group and no metastasis group in cardiac cancer [22]. MDCT can also indicate the situation in other abdominal organs and the peritoneum. Therefore, MDCT imaging can accurately reflect the biological behavior of gastric cancer histopathology. Univariate analysis in our study showed that the 6 indicators of gastric cancer and lymph nodes information on CT images all have a relation to LNM. So we should consider these biological behaviour factors comprehensively in predicting LNM.

There were some other machine-learning methods used in medical studies. The mainly method was artificial neural network (ANN). ANN is considered to be an appropriate method for medical data analysis [23]. Bollschweiler et al applied a single-layer perceptron, which is a kind of ANN, to predict lymph node metastasis in gastric cancer. The accuracy of ANN was 79% [24]. However, the ANN had some disadvantages. ANN's model was prone to overfitting. It required lengthy development and time to optimize. They were more difficult to use in the field because of computational requirements [25]. In consideration of the above reasons, we selected the SVM model instead. The SVM could produce lower prediction error compared to classifiers based on other methods like artificial neural networks [26]. Compared with ANN, SVM may have the same even better predictive ability [27,28]. At present, there are few reports about the application of SVM in gastric cancer lymph node metastasis. As a preliminary study, our results indicate that SVM model has better diagnostic capability for LNM than the traditional LN size criteria. The AUC has achieved a good diagnostic power. With further improvement, SVM may become an effective method to predict lymph node staging of gastric cancer.

## Conclusions

Based on biological behavior information of gastric cancer on MDCT images, SVM model can help diagnose the lymph node metastasis preoperatively.

## Competing interests

The authors declare that they have no competing interests.

## Authors' contributions

XPZ carried out the study design and data acquisition. ZLW carried out the data interpretation and manuscript editing. LT, KC carried out the manuscript drafting for intellectual content. YSS, YG participated in the design of the study and the statistical analysis. All authors read and approved the final manuscript.

## Pre-publication history

The pre-publication history for this paper can be accessed here:

http://www.biomedcentral.com/1471-2407/11/10/prepub
